# Potential therapeutic effect of thymoquinone and/or bee pollen on fluvastatin-induced hepatitis in rats

**DOI:** 10.1038/s41598-021-95342-7

**Published:** 2021-08-03

**Authors:** Amro E. Mohamed, Mohammed A. El-Magd, Karim S. El-Said, Mohamed El-Sharnouby, Ehab M. Tousson, Afrah F. Salama

**Affiliations:** 1 Biochemistry Division, Chemistry Department, Faculty of Science, Tanta University, Tanta, Egypt; 2 Department of Anatomy, Faculty of Veterinary Medicine, Kafrelsheikh University, Kafr El Sheikh, Egypt; 3 Department of Biotechnology, College of Science, Taif University, P.O. Box 11099, Taif, 21944 Saudi Arabia; 4 Zoology Department, Faculty of Science, Tanta University, Tanta, Egypt

**Keywords:** Biochemistry, Histocytochemistry

## Abstract

Hepatitis is one of earlier, but serious, signs of liver damage. High doses of statins for a long time can induce hepatitis. This study aimed to evaluate and compare the therapeutic potential of thymoquinone **(**TQ) and bee pollen (BP) on fluvastatin (F)-induced hepatitis in rats. Rats were randomly divided into: group 1 (G1, control), G2 (F, hepatitis), G3 (F + TQ), G4 (F + BP), and G5 (F + TQ + BP). Single treatment with TQ or BP relieved fluvastatin-induced hepatitis, with best effect for the combined therapy. TQ and/or BP treatment significantly (1) reduced serum levels of alanine aminotransferase, aspartate aminotransferase, alkaline phosphatase, gamma glutamyl transpeptidase, and total bilirubin, (2) decreased malondialdehyde levels and increased level of reduced glutathione, and activities of glutathione peroxidase and catalase in the liver, (3) improved liver histology with mild deposition of type I collagen, (4) increased mRNA levels of transforming growth factor beta 1, nuclear factor Kappa B, and cyclooxygenase 1 and 2, and (5) decreased tumor necrosis factor alpha and upregulated interleukin 10 protein in the liver. These data clearly highlight the ability of TQ and BP combined therapy to cause better ameliorative effects on fluvastatin-induced hepatitis than individual treatment by each alone.

## Introduction

Liver damage constitutes one of the main causes of hepatocellular carcinoma (HCC) that leads to death worldwide^1–3^. Hepatitis is one of earlier signs of liver damage, which is characterized by inflammatory cells infiltration, congestion of blood vessels, and cellular degenerative changes. Hepatitis could be induced by a viral infection, microbial metabolites, metabolic and autoimmune diseases, environmental toxicants, and alcohol and drug abuse^1,3,4^. If hepatitis was left for a long time without suitable treatments, other complicated liver disorders such as fibrosis and cirrhosis would be developed and in advanced cases become risk factors for HCC^5^.

Lipid-lowering drugs statins, including fluvastatin, prevents cholesterol biosynthesis by inhibiting hydroxyl-methyl-glutaryl co-enzyme A (HMG-CoA) reductase activity^6^. Higher doses of statins were reported to induce hepatotoxicity and myotoxicity^7,8^. Rats administrated fluvastatin (24 mg/kg/day) for 7 days suffered from notable hepatotoxicity^7^. Treatment with high doses of fluvastatin disturbs liver function as evidenced by elevation of alanine aminotransferase (ALT) and aspartate aminotransferase (AST) serum levels and induces myopathy as revealed by high serum AST and creatine kinase levels in rats^8^.

Alternative therapeutic strategies using natural products have been traditionally utilized in the treatment of liver damage and cancer^9–13^. Among these medicinal natural products, *Nigella sativa* and its main phytochemical component thymoquinone (TQ) have several health beneficial properties, such as antioxidant, anti-inflammatory, immune-stimulant, anti-bacterial, hypoglycemic, and anti-arthritic activities^14–18^. Due to their potent antioxidant, anti-inflammatory properties, TQ relieved hepatotoxicity induced by carbon tetrachloride (CCl_4_) in mice^19^, and by 2,3,7,8-tetrachlorodibenzo-p-dioxin^20^, ethanol^21^ and gentamicin^22^ in rats by inhibiting oxidative stress, inflammation, and apoptosis. TQ also attenuated chemically-induced liver fibrosis in mice^23^.

The honeybees gather bee pollen (BP) as a supply of nutrients for their hives. Male flowers' reproductive cells produce pollen grains, that contain large amounts of phenolic compounds, phytochemicals, flavonoids, carotenoids, amino acids, minerals, and vitamins^24,25^. However, the exact ingredients of BP differ based on plant types and growth conditions^26^. BP was used for many years as a food supplement, or even as a medicine, to improve health especially for people with cardiovascular diseases^27^. Similar to TQ, BP also had many health-promoting properties including anti-oxidant, anti-inflammatory, anti-cancer, anti-bacterial, and anti-atherosclerotic activities^28^. The potent BP anti-oxidant properties were mainly attributed to its higher content of phenolic compounds^29^. Consumption of BP significantly reduced oxidative stress and increased the activities of antioxidant enzymes in rats^25^. Previous studies reported potent hepatoprotective effect for BP and other apitherapy products with an effect similar to silymarin, the most commonly used drug in the treatment of and/or protection against many liver injuries^30–32^. For this reason, BP and other apitherapy products utilized to attenuate hepatotoxicity induced by CCl_4_^33,34^, carbaryl^24^, and aluminum^35^ in rats.

The scientific gaps of previous studies included a lack of investigations on the therapeutic potential of either TQ or BP on the hepatitis model induced by fluvastatin or any other statins. Searching the available published data, we also did not find researches evaluating the combined effect of TQ and BP on hepatitis or liver injury animal models. It is also important to know whether single or combined treatment of these two natural products can give better improvement for fluvastatin-induced hepatitis. To fill in these scientific gaps, herein we evaluated and compared the efficacy of TQ and BP treatment on hepatitis induced by fluvastatin in rats using biochemical, structural, and molecular approaches.

## Results

### Effect of thymoquinone and/or bee pollen on liver function

Rats administered fluvastatin (hepatitis group) showed significantly (*p* < 0.05) higher levels of liver damage parameters (AST, ALT, ALP, γ-GTP, and total bilirubin) and significantly lower levels of albumin than the control group (Table 1). Treatment with TQ and/or BP restored these parameters to levels comparable to the control group. The highest improvement was observed in the co-treated group (TQ + BP) followed by the TQ-treated group. However, BP-treated group showed less improvement in these parameters, but still significantly different from the hepatitis group. These findings indicate that treatment with TQ and/or BP could ameliorate fluvastatin-induced liver damage with best effect for the co-treated group followed by TQ and then BP group.

**Table 1:** Effect of TQ and/or BP treatment on serum levels of liver damage parameters in fluvastatin-induced hepatitis in rats.

Parameters	Control (G1)	F (G2)	F + TQ (G3)	F + BP (G4)	F + TQ + BP (G5)
ALT (U/L)	39.54 ± 3.64^e^	180.11 ± 7.30^a^	81.2 ± 6.83^c^	109.66 ± 6.57^b^	62.48 ± 4.23^d^
AST (U/L)	137.49 ± 6.71^e^	375.45 ± 10.49^a^	216.06 ± 8.69^c^	252.58 ± 9.52^b^	171.54 ± 7.13^d^
ALP (U/L)	89.37 ± 5.27^e^	281.34 ± 13.01^a^	162.14 ± 7.01^c^	208.26 ± 8.17^b^	111.36 ± 6.11^d^
γ-GTP (U/L)	7.06 ± 0.62^d^	25.33 ± 1.20^a^	15.85 ± 0.64^b^	17.76 ± 0.81^b^	10.36 ± 0.54^c^
Albumin (g/dl)	4.12 ± 0.23^a^	2.05 ± 0.12^d^	3.00 ± 0.13^c^	2.71 ± 0.16^c^	3.59 ± 0.14^b^
Total bilirubin (mg/dl)	0.95 ± 0.07^d^	3.32 ± 0.14^a^	2.12 ± 0.13^bc^	2.44 ± 0.11^b^	1.63 ± 0.09^c^

Data are expressed as mean ± SEM (n = 7/group). Mean values with different superscript letters [a (the highest values) – e (the lowest value)] in the same row are significantly different at (*p* ≤ 0.05). All groups were compared to each other. BP, Bee pollen; G1-5, Groups 1–5; F, Fluvastatin; TQ, Thymoquinone.

### Effect of thymoquinone and/or bee pollen on oxidative stress and antioxidant biomarkers

**Figure 1:**
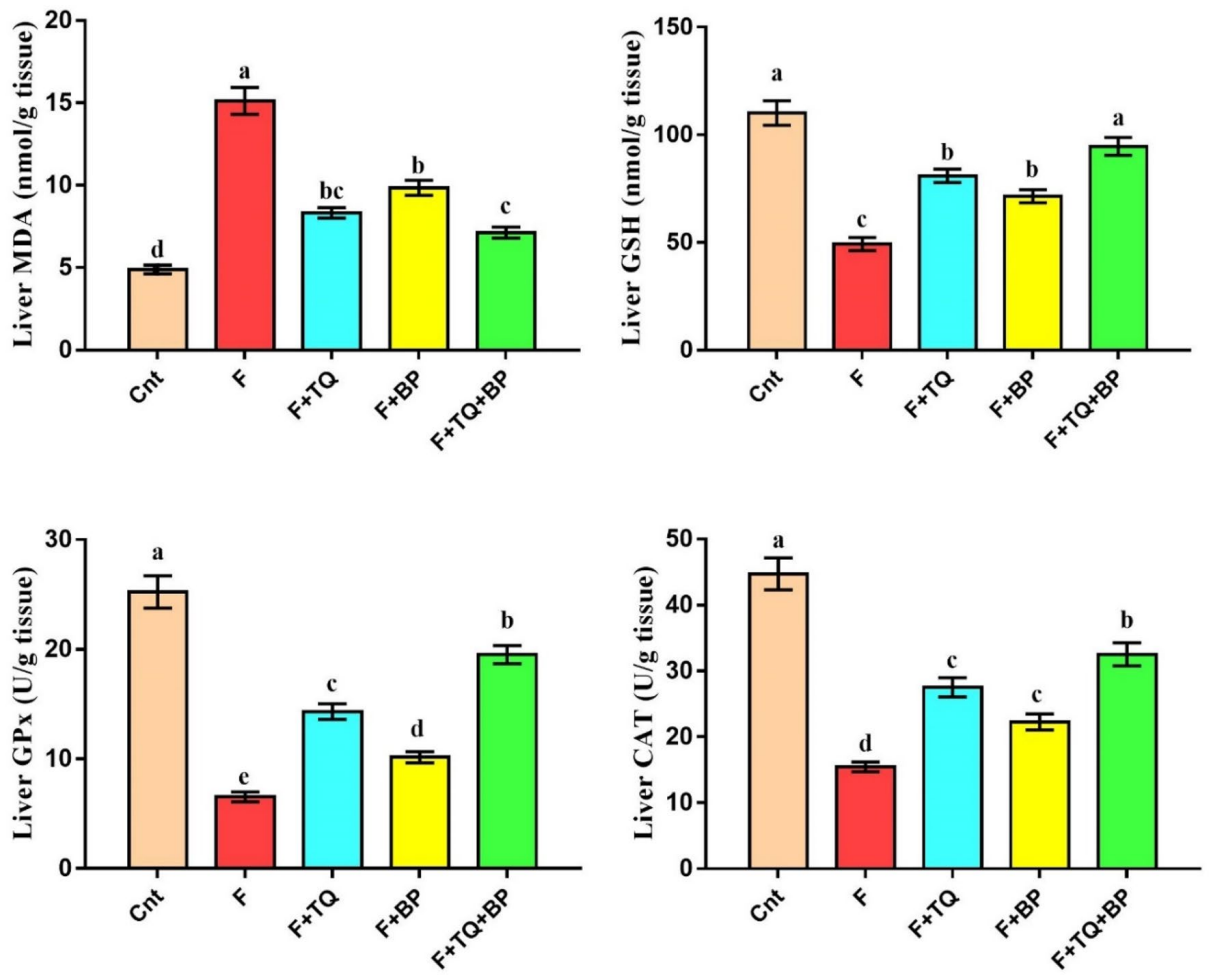
Effect of TQ and/or BP treatment on oxidative stress (MDA) and antioxidant status (GSH, GPx, and CAT) in rat liver. Values are expressed as mean ± SEM (n = 7/group). Columns carrying different letters [a (the highest value) – e (the lowest value)] are significantly different at *p* < 0.05. All groups were compared to each other. Cnt: control group (G1); F, Fluvastatin-treated group (G2); F + TQ, Thymoquinone-treated group (G3); F + BP, Bee pollen-treated group (G4); F + TQ + BP, co-treated group (G5). (GraphPad Prism 8, https://www.graphpad.com/scientific-software/prism/).

Animals treated with fluvastatin (hepatitis group) had a significantly higher MDA level and a significantly lower GSH level and antioxidant activities of GPx and CAT in their livers than control animals (Fig. 1). Administration of TQ and/or BP restored these oxidant/antioxidant biomarkers to levels comparable to the control. Again, among the three treated groups, the co-treated group showed best improvement (lowest MDA and highest GSH, GPx, and CAT) followed by the TQ group (Fig. 1). Also, rats administrated BP had a significant lower MDA and higher GSH, GPx, and CAT than the hepatitis group. These data imply that TQ and/or BP could attenuate oxidative stress damage in the liver induced by fluvastatin through inhibition of the lipid peroxidation MDA biomarker and activation of antioxidant biomarkers with the best effect in the co-treated group.

### Effect of thymoquinone and/or bee pollen on liver histology

**Figure 2:**
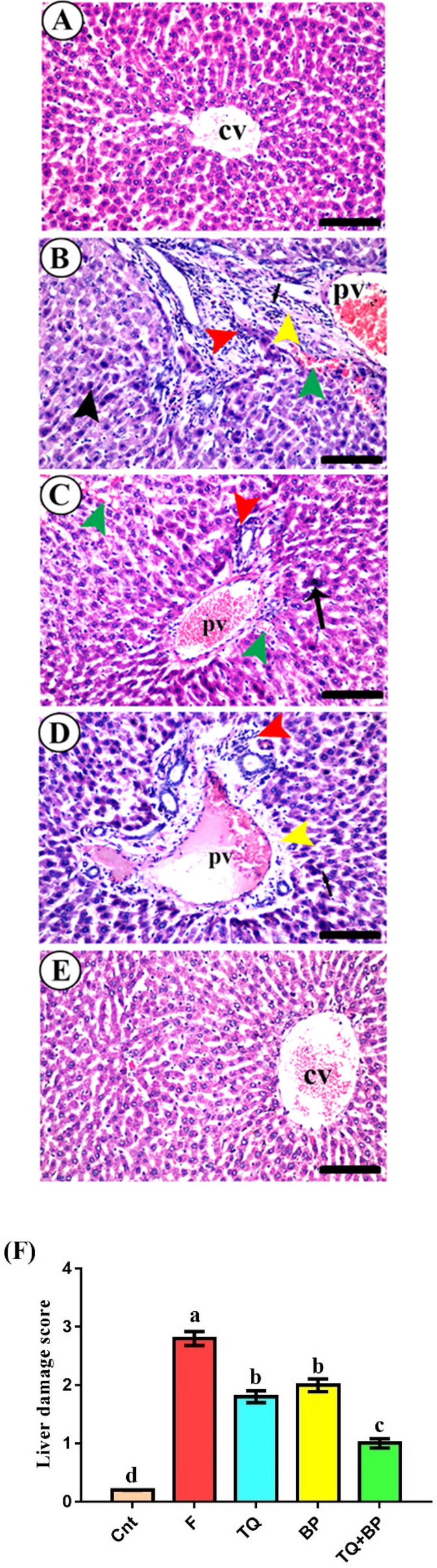
Photomicrographs of rat liver sections stained with H&E in control group (**A**), fluvastatin-treated group (**B**), thymoquinone-treated group (**C**), bee pollen-treated group (**D**); TQ + BP co-treated group (**E**). All labels were explained in the main text. Scale bars = 40 µm (**A**, **E**) and 30 µm (**B**–**D**). (**F**) Liver damage score, values are the mean ± SEM (n = 5/group). Columns carrying different letters (a–d) are significantly different at *p* < 0.05.

No histopathological changes were noticed in the liver of the control group. Livers of these animals showed normal structure with hepatocytes arranged in cords radiating from the central vein (cv) within the parenchyma (Fig. 2A). However, fluvastatin-treated rats exhibited typical histological alterations associated with hepatitis in form of severe congestion in the portal vein (pv), aggregation of numerous inflammatory mononuclear cells (red arrowhead) in the vicinity of the portal vein (Fig. 2B). Some hepatocytes had early signs of necroptosis such as pyknotic nuclei that contained condensed chromatin (black arrowhead, Fig. 2B). Fibrosis in form of aggregation of fibroblasts (arrow) and collagen fibers (yellow arrowhead), as proved later by immunohistochemistry, was also noticed in liver parenchyma especially in the portal area and adjacent to the area of mononuclear cells infiltration (Fig. 2B). Moreover, few extravasated red blood cells were seen in parenchyma around the portal vein (green arrowhead, Fig. 2B). On the other hand, liver sections in TQ and BP treated groups showed partial improvement in liver architecture with better regeneration in the TQ group. The TQ-treated group showed a mild degree of inflammation with moderate congestion in the portal vein (pv) and a very few inflammatory cells infiltration (red arrowhead, Fig. 2C). Also, nuclear pyknosis (arrow) in few hepatocytes and few extravasated blood (green arrowheads) were noticed (Fig. 2C). The BP-treated group exhibited mild congestion in the portal vein (pv) with moderate inflammatory cells infiltration (red arrowhead), slight perivascular edema and fibrosis (yellow arrowhead), and few hepatocytes nuclear pyknosis (arrow, Fig. 2D). The co-treated group showed the best signs of tissue regeneration with nearly similar tissue architecture to the control group, except for the presence of slightly congested central veins (cv, Fig. 2E). The highest liver damage score was found in the F group and was significantly reduced following treatment with the TQ and/or BP with best improvement in the combined treated group (Fig. 2F). We could infer that treatment with TQ and/or BP ameliorated liver histological damage induced by fluvastatin, with best improvement for the co-treated group.

### Effect of thymoquinone and/or bee pollen on COLI immunostaining

**Figure 3:**
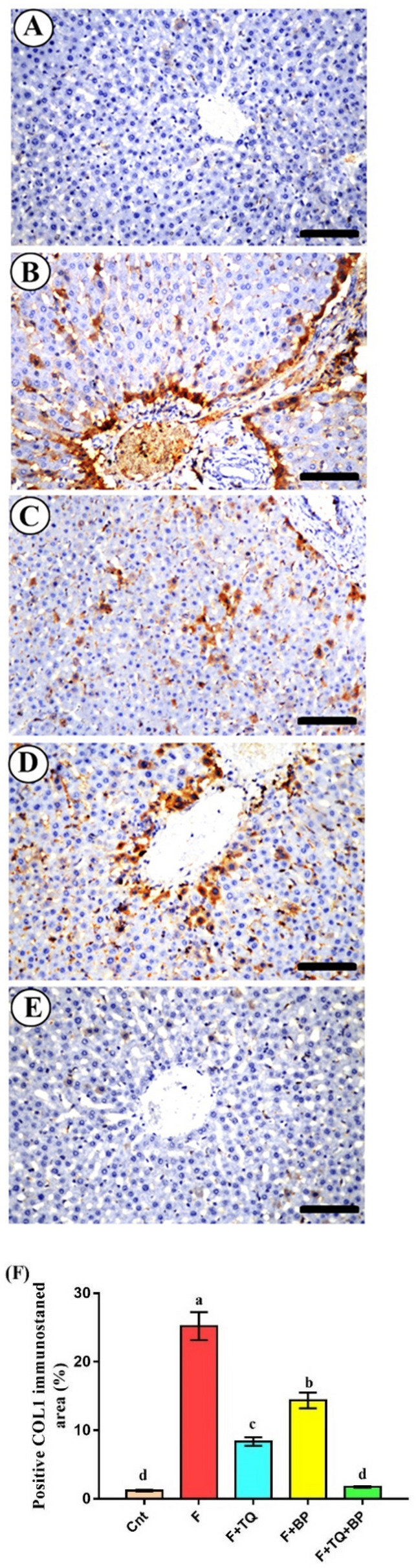
(**A**–**E**) Photomicrographs of liver sections immune-stained with COL1 in control group (**A**), fluvastatin-treated group (**B**), thymoquinone-treated group (**C**), bee pollen-treated group (**D**); TQ + BP co-treated group (**E**). Scale bars = 40 µm. (**F**) Quantification of COL1 positive immune staining. Data were presented as mean positive stained area (%) ± SEM (n = 7/group). Columns with different lower-case letters [a (highest value) – d (lowest value)] are significantly different at *p* < 0.01. All groups were compared to each other.

Liver parenchyma of the control group showed very little deposition of collagen I (COLI, Fig. 3A). In contrast, liver sections of the fluvastatin-treated group exhibited an abundant positive COL1 immuno-stained area in the portal area (Fig. 3B). COLI positive area was significantly higher in the fluvastatin-treated group (25.20 ± 2.03%) than the control group (1.20 ± 0.11%, Fig. 3F). This positive area was significantly reduced in livers of rats treated with either TQ (8.36 ± 0.62%, Fig. 3C,F) or BP (14.36 ± 1.15%, Fig. 3D,F). The co-treated animals showed very little COLI immunostaining (1.73 ± 0.13%) with no significant difference from the control group (Fig. 3E,F). These results suggest induction of liver fibrosis by fluvastatin and a probable anti-fibrotic effect for TQ and BP.

### Effect of thymoquinone and/or bee pollen on expression of inflammatory genes/proteins

**Figure 4:**
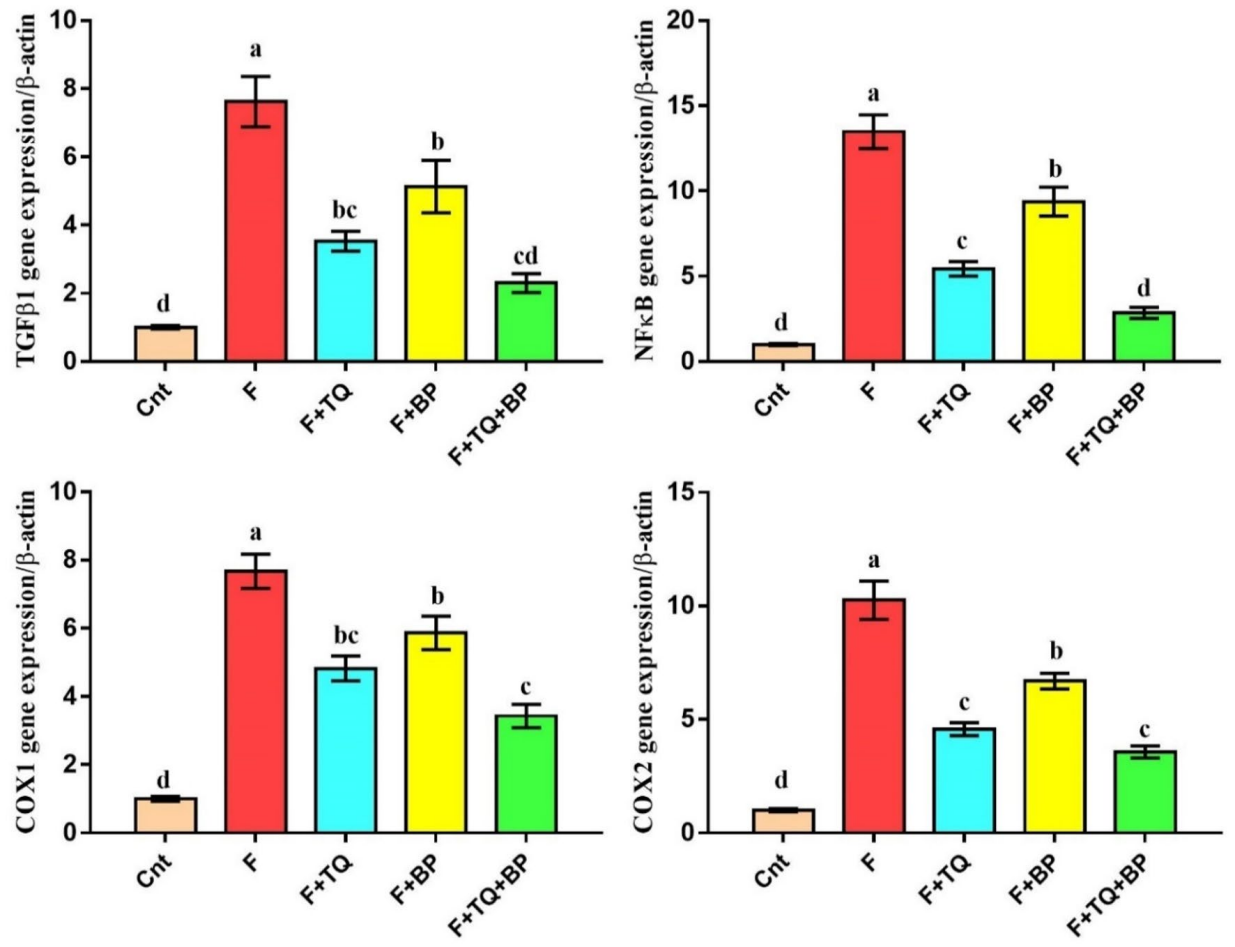
Real-time PCR analysis shows changes in the relative expression of *TGFβ1*, *NFκB*, *COX1*, and *COX2* genes relative to the housekeeping *β-actin* gene. Gene expression fold changes were presented as mean ± SEM (n = 7/group). Columns carrying different letters [a (the highest value) – d (the lowest value)] are significantly different at *p* < 0.05. All groups were compared to each other.

Real-time PCR (qPCR) was applied to determine inflammatory genes, TGFβ1, NFkB, COX1, and COX2 expression in rat liver following different treatments. The obtained results revealed a significant increase in the expression of these genes in the fluvastatin-treated group as compared to the control group (Fig. 4). Treatment with TQ and/or BP significantly downregulated these genes, with lowest expression in the co-treated group, followed by the TQ group, as compared to the fluvastatin-treated group (Fig. 4).

**Figure 5:**
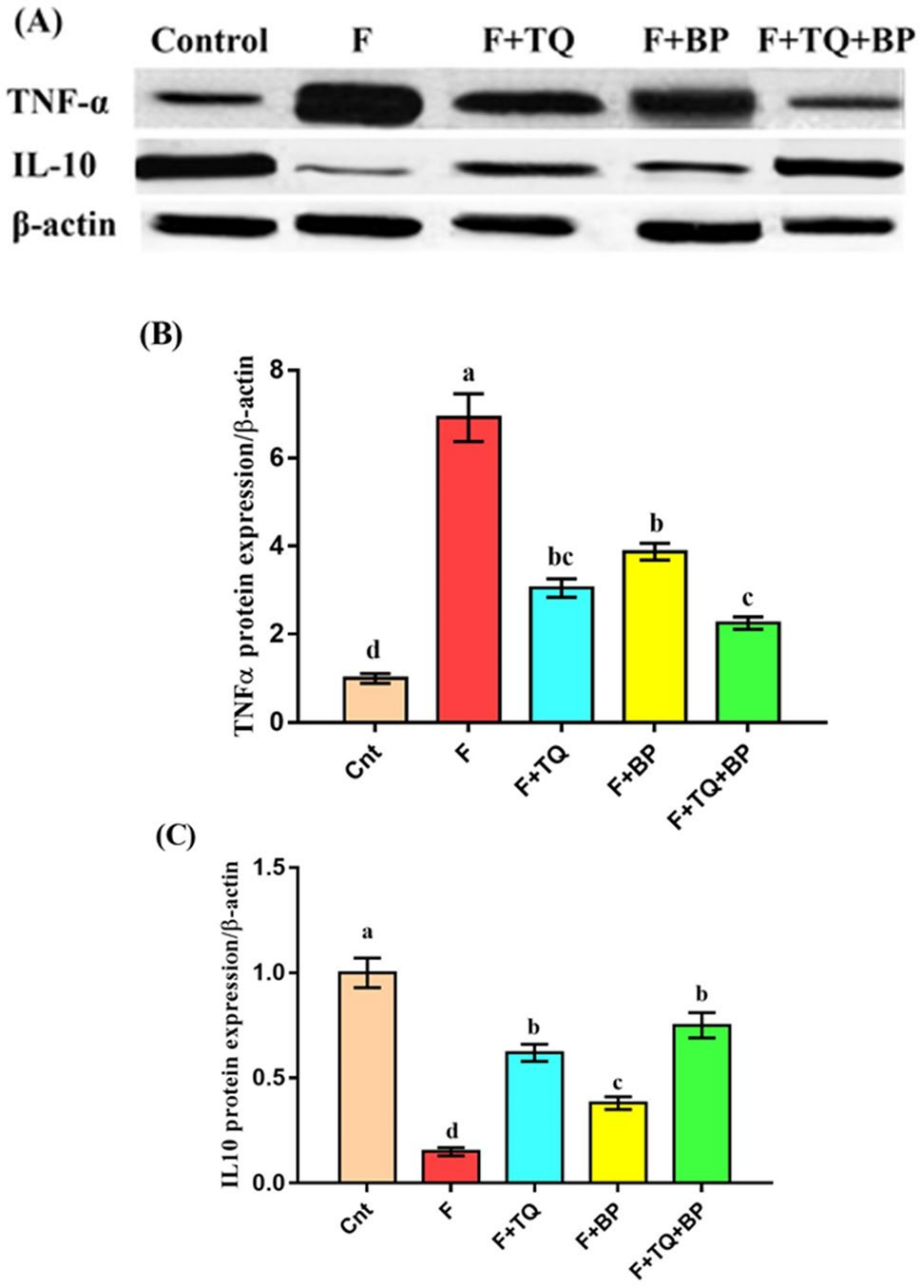
Western blot analysis shows changes in TNFα and IL10 protein expression relative to β-actin protein (control). (**A**) TNFα and IL10 protein bands. (**B** and **C**) Band quantification of TNFα and IL10 protein (ImageJ, https://imagej.nih.gov/ij/index.html). Data presented as fold change (mean) ± SEM (n = 7/group). Columns carrying different letters [a (the highest value) – d (the lowest value)] are significantly different at *p* < 0.05. All groups were compared to each other.

Western blot analysis was performed to evaluate changes in the expression of the inflammatory protein TNFα and the anti-inflammatory protein IL10 in the liver. Fluvastatin-treated rats had a significantly (*p* < 0.001) higher TNFα expression and a significantly (*p* < 0.001) lower IL10 expression relative to the control group (Fig. 5 and Fig. S1). Treatment with TQ and/or BP restored TNFα and IL10 to levels comparable to the control group, with best results for the co-treated group (which showed lowest TNFα and highest IL10 expression) followed by TQ and BP group (Fig. 5). These molecular-based data confirm the induction of hepatitis by fluvastatin and the ameliorative anti-inflammatory effects of TQ and BP.

## Discussion

A large body of studies investigated the ameliorative effect of TQ and BP each alone on different animal models of hepatotoxicity. However, to date little, or even no, information is available in the literature addressed whether TQ and/or BP could relieve fluvastatin-induced hepatitis. To the best of our knowledge, this may be the first study to report that the administration of TQ and/or BP ameliorated hepatitis induced by fluvastatin with best improvement when the two natural products were given together. This overall conclusion was reached depending on our obtained data that animals treated with TQ and/or BP had lower serum levels of liver damage parameters, decreased oxidative stress and histological damage degree, mild deposition of COLI, and decreased expression of inflammatory genes/proteins in their livers.

In the present study, rats orally administrated fluvastatin had a significant elevation in serum levels of liver damage parameters (ALT, AST, ALP, γ-GTP, and total bilirubin) with a significant reduction in the albumin level. This infers that treatment with fluvastatin can induce liver damage. Consistent with our findings, other studies also reported that fluvastatin administration caused hepatocellular damage with a significant increase in AST, ALT, ALP and γ-GTP activities^7,8,36^. Statins are metabolized by the liver and this could be the cause of hepato-toxicity and elevation of liver damage parameters following long-lasting treatment^37^. In humans with familiar hypercholesterolemia, statins, including fluvastatin, are very important hypocholesterolemic drugs that also decrease the severity of the associated cardiovascular diseases, thereby reducing the death rate. However, unlike animals which showed clear hepatitis, the most common side effect for statins in humans is the myopathy, which is characterized by rhabdomyolysis^38^. Cokça, et al.^39^ reported a case of female suffers from severe liver and muscle injury following taken an overdose of statins. Some patients taken the allowed dose also showed mild symptoms of liver injury following long-lasting treatment with statins^40,41^. Thus, it is likely that statins dose and duration is more essential determinants of hepato- and myo-toxicity than hypocholesterolemia^42^. Our results also showed that TQ and/or BP treatment could ameliorate hepatitis induced by fluvastatin and restored liver function. The two natural products restored the elevated liver enzymes (ALT, AST, γ-GTP, and ALP) to levels comparable to the control group, with best improvement for the combined therapy. Additionally, the two natural products elevated albumin serum levels, which proved the recovery of liver function and reverted bilirubin to normal levels. Similarly, other studies showed that supplementation with TQ^4,22,43^ or BP^33^ induced hepatoprotective and therapeutic effects against different models of liver injury.

Oxidative stress is one of the most common causes of liver injury. Our findings revealed that fluvastatin administration induced a significant elevation in hepatic MDA level and a significant reduction in hepatic GSH concentration and activities of GPx and CAT antioxidant enzymes. Increased MDA levels in the fluvastatin-treated group could increase lipid peroxidation which damaged lipid in cellular components, especially the cell membrane. This could explain leakage of liver damage parameter from liver to the circulation^1^. Free radicals overproduction and reactive oxygen species (ROS) are the main causes for oxidative stress and although hepatocytes have a more potent endogenous antioxidant system, they are prone to damage by these harmful elements when they exceed cells response to stress. Treatment with TQ and/or BP decreased hepatic oxidative stress induced by fluvastatin (as indicated by lower MDA level) and increased hepatic antioxidant status (higher GSH level and GPx and CAT activities), with best effect for the co-treated group. TQ has promising radicals scavenger activities enabling it to reduce oxidative damage and improve activities of liver endogenous antioxidant enzymes in animal models of liver injury induced by CCl_4_^19^, ethanol^21^, or gentamicin^22^. BP has potent antioxidant activities due to high contents of phenolic compounds, phytochemicals, and flavonoids, especially quercetin, that prevent free radical production and increase the activity of antioxidant enzymes^24,25,29,44^. Consistent with our results, other studies also found an ameliorative effect for BP, through induction of antioxidant status and inhibition of oxidative stress, on hepatotoxicity induced by CCl_4_^33,34^ and carbaryl^24^ in rats. Moreover, BP induced a more potent hepatoprotective effect than propolis on cisplatin-induced hepatotoxicity in mice^45^.

At a histological level, fluvastatin also induced typical signs of inflammation as evidenced by the portal and central veins congestion and inflammatory mononuclear cells infiltration. Similar histopathological alterations following treatment with fluvastatin^46^ or rosuvastatin^47^ were also observed. However, unlike this study, we did not find vacuolar degeneration in hepatocytes but rather we found a slight degree of hemorrhage in the liver parenchyma. We also found an early sign of necroptosis where nuclei of some hepatocytes become pyknotic. Chromatin condensation was considered as an early sign of necroptosis in cells^48^. Necrosis was not reported in the liver of rat treated with fluvastatin^46^, however, it was noticed in both the liver of rat administrated rosuvastatin^47^ and in skeletal muscles of HMG-CoA reductase (the main target for statins) knockout mice^49^. Animals treated with TQ or BP showed partial regenerative changes in the liver with slightly better improvement in the TQ group. However, those treated with TQ and BP together had normal liver histology. Thus, treatment with TQ and/or BP restored liver histological damage induced by fluvastatin to level comparable to that of the normal animals, with best results for the combined group. Similarly, administration of either TQ^19,21,22^ or BP^33,34,45^ improved liver histology in other chemically-induced liver damage models.

One of the current hot topic in the setting of liver diseases is the diagnosis of drug-induced liver damage (DILI) and, of particular interest, is the differential diagnosis between DILI and autoimmune hepatitis (AIH), especially in the form of DILI with autoimmune features named as DILI-AIH. This has relevant clinical and therapeutic implications since the specific treatment of DILI-AIH like is based on corticosteroid treatment^50^. The typical histological changes associated with AIH included interface hepatitis, lobular hepatitis and centrolobular necrosis^50^. Since histological evaluation described in the present study clearly excludes histological changes consistent with autoimmune hepatitis, the fluvastatin (F)- induced hepatitis is not a DILI-AIH like and therefore does not require corticosteroid treatments.

Our histological findings supported by results of COLI immunostaining revealed induction of fibrosis in the liver following treatment with fluvastatin. A significantly higher positive COLI immunostaining was detected in the portal area. Most cases of acute hepatitis induced by chemicals or toxins would be transformed into chronic hepatitis, which further resulted in hepatic fibrosis if left without treatment^51^. Treatment with TQ reduced liver fibrosis as revealed by a reduction of COLI immunostaining^52^. The antifibrotic effect of TQ was also reported by Ghazwani, et al.^52^ who attributed this effect to TQ ability to downregulate fibrosis-related genes *COL1*, *TGFß1*, and α-smooth muscle actin (*α-SMA*) in a mouse model of CCl_4_-induced liver fibrosis. Bai et al.^23^ also showed that TQ could decrease liver fibrosis triggered by thioacetamide by targeting COLI expression in the liver. Unlike TQ, PB exerted less antifibrotic effect, however, fibrosis was significantly reduced as compared to the fluvastatin-treated group. A similar mild antifibrotic effect of BP on a rat model of CCl_4_-induced liver fibrosis was also reported^33^. However, another study reported potent anti-fibrotic and anti-necrotic effects for BP against cisplatin-induced liver injury in mice^45^.

In addition to the typical histological lesion of hepatitis seen in the fluvastatin-treated group, hepatitis was confirmed on molecular levels. We found a significantly higher expression of inflammatory genes (*TGFβ1*, *NFkB, COX1*, and *COX2*) and TNFα protein and a significantly lower expression of the anti-inflammatory IL10 protein in the fluvastatin-treated group as compared to the control group. High mRNA levels of the epithelial-mesenchymal transition marker *TGFβ1* could explain the occurrence of fibrosis in fluvastatin-treated animals^53^. Additionally, the elevation of TNFα protein, *NFkB* and its downstream target *COX2* gene could also be associated with chronic inflammation and fibrosis^54^. Persistent high NFkB mRNA and protein levels in chronic hepatitis is the main inducer of liver cancer^55^. Our data showed that animals treated with TQ and/or BP had a lower expression of these inflammatory markers and a higher expression of IL10. This suggests an ameliorative effect of these two natural products, with best effect for the combined therapy, on the fluvastatin -induced hepatitis. Similarly, TQ has been reported to decrease TNFα, NFκB, and COX2 levels associated with many inflammatory conditions [Reviewed by^56^]. Bai et al.^23^ also demonstrated that TQ anti-fibrotic effect on the liver was mediated, at least in part, via the inhibition of TGFβ. Furthermore, TQ potentially reduced inflammation through targeting COX1,2^57^. The anti-inflammatory activity of BP and its main component quercetin was mediated by targeting COX1,2 and subsequent inhibition of the NFκB pathway^58^.

Statins are widely used for lowering total cholesterol and reducing the risk of a heart attack or stroke. Introduced 40 years ago, statins rarely cause liver disease in humans, they only induce a mild increase in liver enzymes^38,39,41^. On rare occasions, they may induce a larger increase and on those occasions a different statin should be used^42^. Among the seven statins used in clinical practice, fluvastatin is actually one of the least used. However, no data are available in the literature regarding the side effect of fluvastatin overdose for a long time. For this reason, we used an overdose (75 mg/kg) a triple of the dose of previous existing studies^7^ to evaluate the possible side effect on the liver. If the same adverse effects were clinically proven in humans, hypercholesteremic patients should avoid fluvastatin overdose.

## Conclusions

To the best of our knowledge, this is the first report to demonstrate that treatment with TQ and/or BP natural products antagonizes the detrimental effects of fluvastatin on rat liver. TQ and BP co-treatment gave better therapeutic potential than each alone. The findings offer new insight into the mode of fluvastatin-induced hepatitis and propose the use of the two natural products TQ and BP together as a promising feed additive to decrease inflammation and oxidative stress induced by fluvastatin and probably other statins. They also could be used as a suitable alternative in the treatment of hepatitis in patients, however further preclinical and clinical studies are required to warrant their safety and efficacy.

## Materials and methods

### Chemicals and natural products

Fluvastatin (Lescol 80 mg tablets) was obtained from (Novartis Pharmaceuticals, Spain). TQ (164.20 molecular weight, 99% purity) was purchased from Sigma-Aldrich (Sigma Chemical Co, USA, CAS Number: 490–91-5). Honeybee pollens (BP) were obtained from local beekeepers in a powder form. The BP source in a particular area is determined by the type of vegetation available (which also varied according to optimal temperatures, and soil texture, pH, and drainage), growing degrees, and the duration of its flowering period. These pollens were gathered by Egyptian bees from plant flowers growing nearby the hives in Tanta, in the north of Delta, Egypt. A suspension of 280 g BP dissolved in 10 ml distilled water was prepared as previously described^45^. Following overnight storage, centrifugation (10,000 rpm/10 °C/45 min), and filtration of supernatant, the obtained filtrate was frozen (− 20 °C) until use. All other chemicals and reagents were of the highest purity available.

### Experimental design

Animals were handled and treated according to guidelines for experimental animals uses in research which was approved by Ethical committee at the Faculty of Science, Tanta University, with ethical approval license (Rec-Sci-Tu-0417). All methods are reported in accordance with ARRIVE guidelines. Fifty adult female Sprague Dawley rats (200 ± 20 g) were obtained from the National Research Center (NRC, Cairo, Egypt), housed under standard environmental conditions (temperature 23 ± 2 °C, humidity of 54 ± 2% and 12 h/12 h light/dark cycle). Rats were randomly divided into five groups (n = 10). In group 1 (G1, control), rats received distilled water. In G2-G5, animals were orally administered with fluvastatin (75 mg/kg body weight/day) for 10 days. The dose of fluvastatin was chosen based on a previous study^46^ and was also confirmed by a pilot study using four different doses (25, 50, 75, and 100 mg/kg/day) for 5, 10, and 20 days. Even, another study used a lower dose of fluvastatin at 24 mg/Kg/day for a shorter time (7 days), we did not find a notable hepatotoxic effect before our selected dose and duration. In G2, (hepatitis group), animals were treated only with fluvastatin for 10 days and left without treatment for a further 10 days. In G3, animals were orally administered with fluvastatin for 10 days and then were orally treated with TQ at a dose of 20 mg/kg/day^59^ from D11 to D20. In G4, animals were treated with fluvastatin for 10 days and then were orally administered with BP at a dose of 400 mg/kg body weight/day^34^ from D11 to D20. In G5, animals treated with fluvastatin for 10 days and then treated with both TQ and BP with similar doses and duration as in G3 and G4. All groups were treated by gavage.

At the end of the experiment (D21), rats were anesthetized by 500 µL of Ketamine-Xylazine intraperitoneal injection (100 and 20 mg/kg body weight, respectively) and blood samples were collected from eye venous plexus in plain tubes. Sera were obtained by centrifugation for the determination of biochemical parameters as previously detailed^2^. After euthanization by exsanguination, livers were immediately excised and divided into three portions; the first portion was stored in − 80 °C freezer (for qPCR and western blot assays), the second portion was homogenized (for biochemical assay), and the third portion was fixed in 10% formalin (for histological investigation).

### Biochemical analysis

Liver damage enzymes [aspartate transaminase (AST), alanine transaminase (ALT), alkaline phosphatase (ALP)] were determined in serum using commercially available kits (Diamond- Diagnostics, Egypt). Gamma glutamyl transpeptidase (γ-GTP), albumin, and total bilirubin were determined in serum using commercially available kits (BioMed-Diagnostics, EGY-CHEM, Egypt). Liver homogenates were prepared as previously described^35^. The levels of lipid peroxidation marker malondialdehyde (MDA) and reduced glutathione (GSH) as well as the activities of the antioxidant enzymes glutathione peroxidase (GPx) and catalase (CAT) were determined in liver homogenates using commercially available kits (Biodiagnostics, Egypt) and as previously detailed^60,61^.

### Histopathological examination

Fixed liver specimens were dehydrated in alcohol, cleared in xylene, embedded in paraffin, sectioned at 5 µm thickness, and stained by hematoxylin and eosin. The liver damage score involved portal and central veins congestion, mononuclear cells infiltration, blood extravasation, hepatocyte nuclear pyknosis, and fibrosis. These histopathological alterations were determined in 10 randomly chosen, non-overlapping fields at a magnification power of 400 X per each specimen. The damage score was set as follows: 0 (no lesion), 1 (mild), 2 (moderate), and 3 (severe)^20^.

### Immunohistochemical examination

Immunostaining of liver specimens was performed as previously described^62,63^. In brief, sections were first incubated with 5% fetal calf serum (to block nonspecific binding), and then with anti-collagen I (COLI) primary mouse monoclonal antibody (1:300 dilution, Santa Cruz Biotechnology, USA, Cat # sc-59772) overnight at 4 °C. Subsequently, sections were incubated with secondary biotinylated mouse antibodies (1:1000, Dako, CA, # K4003) for 1 h, followed by incubation with streptavidin–biotin peroxidase conjugate (1:100, Vector Laboratories, Burlingame, CA) and with diaminobenzidine chromogen to initiate color development. Finally, sections were counterstained with Harris 's modified hematoxylin. COLI positive staining area (%) was quantified at magnifications of X400 by an image analyzer (Leica Q 500 DMLB, Leica, UK). We examined 50 specimens (n = 10/group). The number of fields per each specimen was 20 fields (4 different non-overlapping fields X 5 different sections).

### Real-time PCR

Relative hepatic expression of inflammation related genes (TGFβ1, NFκB, COX1, and COX2) was determined using real-time PCR (qPCR). Total RNA was extracted from liver tissues using a commercially available kit (GeneJET RNA Purification Kit, Thermo Scientific, # K0731, USA) as previously detailed^64^. Before reverse transcription, all isolated RNA samples were quantified by a Nanodrop (Quawell, USA). Reverse transcription of RNA into cDNA was achieved using a commercially available kit (Thermo Scientific, #EP0451, USA). A total qPCR reaction mixture with a volume of 20 µl was prepared. This mixture contained cDNA, QuantiTect SYBR Green qPCR Master Mix, and specific primers designed by the Primer 3 web-based tool based on published rat sequences and previous publications (Table 2). Thermal conditions in StepOnePlus qPCR thermal cycler (Applied Biosystem, USA) included one cycle of initial denaturation at 94 °C for 4 min, followed by 40 cycles of amplification each cycle contained denaturation at 94 °C for 40 s, annealing at 60 °C for 30 s, and extension at 72 °C for 30 s. β actin was used as an internal control. Fold changes of relative expression were determined and the melting curve condition was set as previously described^65–67^.

**Table 2:** Forward and reverse primers sequence for real time PCR.

Gene	Forward primer (/5–/3)	Reverse primer (/5–/3)
TGFβ1	AAGAAGTCACCCGCGTGCTA	TGTGTGATGTCTTTGGTTTTGTCA
NFκB	CCTAGCTTTCTCTGAACTGCAAA	GGGTCAGAGGCCAATAGAGA
COX1	CCCAGAGTCATGAGTCGAAGGAG	CAGGCGCATGAGTACTTCTCGG
COX2	GATTGACAGCCCACCAACTT	CGGGATGAACTCTCTCCTCA
β-actin	ACCCACACTGTGCCCATCTA	CGTCACACTTCATGATG

### Western blot

The expression of both TNFα and IL10 proteins were detected in liver tissues of all groups using western blot assay. This assay was done as previously described^66^. Briefly, liver specimens were lysed using RIPA lysis buffer. The obtained proteins were separated on SDS-PAGE gels and transferred to a polyvinylidene difluoride membrane (PVDF). PVDF membranes were separately incubated with the following mouse monoclonal primary antibodies: anti-TNFα (1:100 dilution, Santa Cruz Biotechnology, Cat # sc-52746), anti-IL10 (1:200 dilution, Santa Cruz Biotechnology, Cat # sc-365858), and anti-β-actin (housekeeping, 1:200 dilution, Cat # sc-47778). PVDF membranes were then incubated for 1 h at room temperature with horseradish peroxidase (HRP)-conjugated goat anti-rabbit secondary antibody (1:2000 dilution, Cell Signaling Technology, Beverly, MA, USA). The color was developed using tetramethylbenzidine (Sigma) and the band density was quantified by ImageJ software.

### Statistical analysis

Normal distribution of data was tested by the Kolmogorov–Smirnov normality test, followed by the Levene test for variance equivalent. One-way analysis of variance was used to assess significant differences among groups using GraphPad Prism 8 (GraphPad Software, Inc., La Jolla, CA, USA). Tukey test was used to compare all groups with each other and to show the significant effect of different treatments. Data were expressed as mean ± standard error of mean (SEM). Significance was set at *p* < 0.05.

### Institutional Review Board statement

 The study was conducted according to the guidelines of the Declaration of Helsinki and approved by Ethical committee at the Faculty of Science, Tanta University, with ethical approval license (Rec-Sci-Tu-0417).

## Supplementary Information

Supplementary Information.

## Data Availability

The data presented in this study are available on request from the corresponding author.
