# Supplementary figures and images for: Potential therapeutic effect of thymoquinone and/or bee pollen on fluvastatin-induced hepatitis in rats
Source: Sci Rep. 2021 Aug 3;11:15688. doi: 10.1038/s41598-021-95342-7 (PMC8333355; doi:10.1038/s41598-021-95342-7)

**Supplementary file**

**
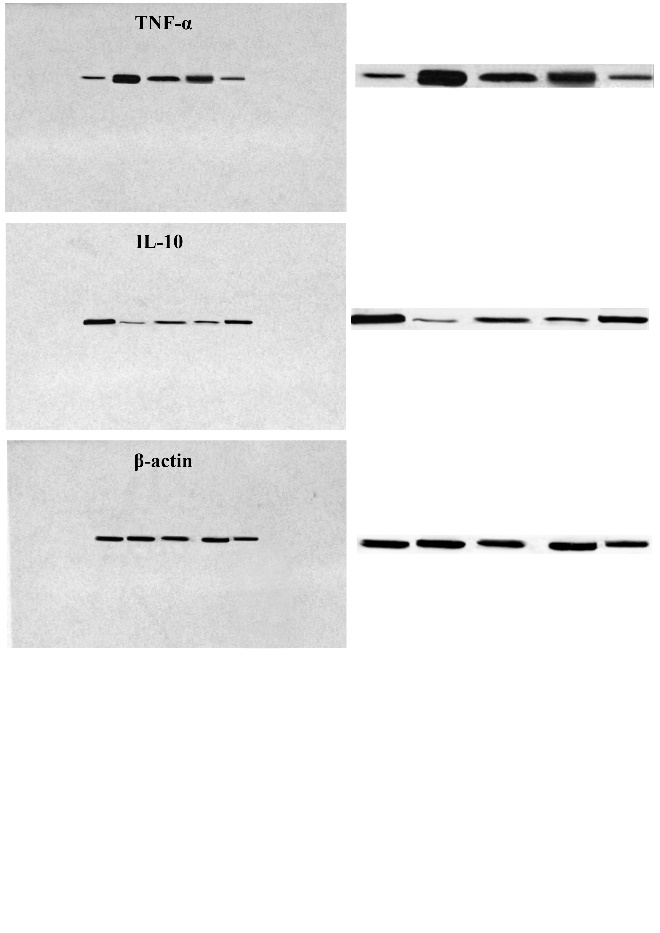
**

Fig.S1: Whole mount western blot.

Supplement: Supplementary file 1 — Supplementary Information. [file 41598_2021_95342_MOESM1_ESM.docx]
